# Professional clinical guidelines for rare diseases: methodology

**DOI:** 10.1186/1750-1172-7-S2-A12

**Published:** 2012-11-22

**Authors:** Odile Kremp, Patrice Dosquet, Ana Rath

**Affiliations:** 1 Orphanet, Inserm, Paris, France; 2 General Directorate for Health, Ministry of Health, Paris, France

## 

Guidelines are systematically developed statements to assist practitioner and patient decisions about appropriate health care for specific clinical circumstances[1]. They concern often complex diagnostic or therapeutic strategies with several possible interventions at each step, where each intervention has to be assessed and compared with other possible interventions at short and long term for efficacy, effectiveness, efficiency (optional), side effects, quality of life, acceptability, benefits/risks ratio. For common diseases, the methodology and the different steps of the guidelines development process have been established and apply the principles of evidence-based-medicine. Developing guidelines for rare diseases (RD) is more difficult, because of the lack of sound evidence, surveys of clinical practices, data about patients’ opinion and ethical reflexions [2].

During the 1st French national plan for rare diseases, HAS (French National Authority for Health) was mandated to define a method to develop guidelines for RD - which are called PNDS (National Diagnostic and Treatment Protocols) in France - and coordinate their writing with the RD centres of reference [3]. A PNDS constitutes a best practice reference document for healthcare professionals in charge of rare disease patients. Based on it, HAS drawn up a “List of Procedures and Services” (LAP), which includes all of the diagnostic tests, drugs, medical devices and services that appear justified in providing care to a patient. From 2005 to now, only 47 PNDS were published and 8 are on-going, because the development method is complex and expertise is scarce; 135 new PNDS projects are pending. In the 2nd French plan for rare diseases it was proposed to define a simplified method for developing the PNDSs, in order to speed up their production, by using the protocols already developed by the RD reference centres, incorporating and adapting recommendations devised by foreign groups of experts when appropriate; and setting criteria for prioritisation of the PNDS production. This process is on its way.

Orphanet has launched the production of emergency guidelines, written by centres of reference in collaboration with emergency department practitioners, patient organisations, and validated by a special reading group with members of emergency medicine learned societies. They include a short description of the disease, recommendations for immediate care, transport and orientation, before getting to the emergency ward, recommendations for the emergency ward (complications to be looked for, diagnostic and therapeutic particularities, drugs interactions, anaesthesia), recommendations for patient’s and family’s comfort, list of contacts 24h/24 [4].
